# Extending limits: the longest recorded duodenojejunal bypass implant

**DOI:** 10.1055/a-2371-1133

**Published:** 2024-08-13

**Authors:** Jan Kral, Pavel Drastich, Manoel P. Galvão, Katerina Knotkova, Martin Haluzik, Evzen Machytka

**Affiliations:** 1 Department of Hepatogastroenterology, Institute for Clinical and Experimental Medicine, Prague, Czech Republic; 2 60568 Department of Internal Medicine, Charles University Second Faculty of Medicine, Prague, Czech Republic; 3 Orlando Health Weight Loss and Bariatric Surgery Institute, Orlando, United States; 4 Department of Diabetes, Diabetes Centre, Institute for Clinical and Experimental Medicine, Prague, Czech Republic

A 59-year-old patient with type 2 diabetes, dyslipidemia, and hypertension underwent the placement of a duodenojejunal bypass (EndoBarrier; GI Dynamics, Boston, Massachusetts, USA). At the time of the endoscopy procedure, the patient weighed 110 kg and was 172 cm tall, resulting in a body mass index (BMI) of 37.3 kg/m ^2^ . The procedure, performed under general anesthesia, concluded without complications. Postoperatively, the patient was prescribed 40 mg of proton pump inhibitors (PPIs) twice daily and advised to avoid fiber. Early side effects included daily nausea (five to eight episodes), and epigastric pain was managed with antiemetics and ongoing PPI use. One month later, an endoscopy confirmed the duodenojejunal bypass was correctly positioned but revealed ulcers at the deviceʼs sharp edges. Despite initial challenges, symptoms improved significantly within three weeks. After 12 months, the patient had lost 30 kg, reducing their (BMI) to 27.3 kg/m ^2^ . The patient chose to keep the bypass despite the conclusion of the study and against medical advice about potential risks.

Over the years, the patient maintained a stable weight of 80 kg and glycemic control with an HbA1c of 41 mmol/mol, managed on 20 mg of PPI and 1000 mg of metformin daily.

Eight years later the diagnostic gastroscopy revealed a torn upper part of the duodenojejunal bypass and a reduced sleeve with the surrounding mucosa irritated. This prompted a planned extraction procedure under general anesthesia, which proceeded without major complications. Following the removal of the duodenojejunal bypass, only minor tears in the esophagus and small ulcers were detected. The patient was discharged the next day without complications and remained stable at a follow-up 1 month later ( Video 1 ).

Extending limits: the longest recorded duodenojejunal bypass implant shows improved body mass index and glycated hemoglobin after 96 months.Video 1

Despite various challenges noted in studies, the duodenojejunal bypass demonstrates significant potential for weight loss and improved management of type 2 diabetes. Our case study features the longest-known implanted duodenojejunal bypass, which maintained its effectiveness even after the upper part was damaged and the sleeve shrank from the original 60 cm to 9.6 cm ( Fig. 1 , Fig. 2 ). This likely contributed to sustained weight management and the remission of type 2 diabetes. Although the device removal can be challenging, the procedure in this instance proceeded smoothly. Nevertheless, technical improvements are essential to reduce these complications and enhance patient safety 1 2 3 .

**Fig. 1:**
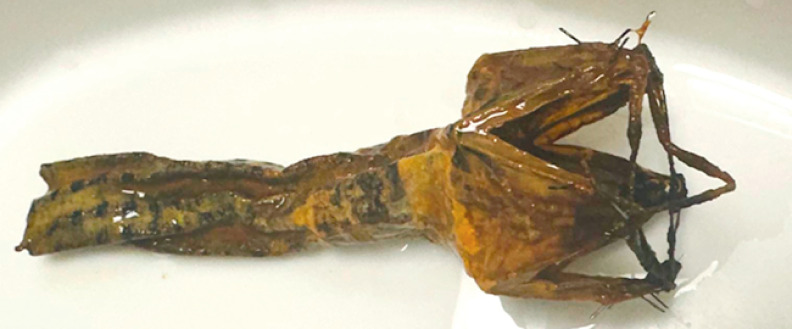
Extracted device I.

**Fig. 2:**
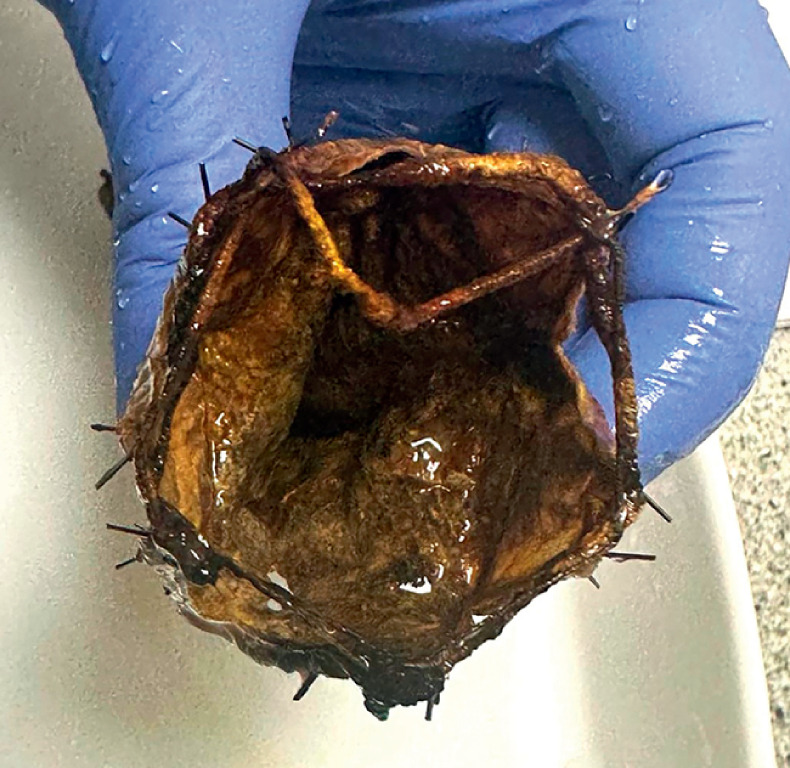
Extracted device II.

Endoscopy_UCTN_Code_TTT_1AO_2AD

## Notes

Endoscopy E-Videos https://eref.thieme.de/e-videos
*E-Videos* is an open access online section of the journal *Endoscopy* , reporting on interesting cases and new techniques in gastroenterological endoscopy. All papers include a high-quality video and are published with a Creative Commons CC-BY license. Endoscopy E-Videos qualify for HINARI discounts and waivers and eligibility is automatically checked during the submission process. We grant 100% waivers to articles whose corresponding authors are based in Group A countries and 50% waivers to those who are based in Group B countries as classified by Research4Life (see: https://www.research4life.org/access/eligibility/ ). This section has its own submission website at https://mc.manuscriptcentral.com/e-videos .
